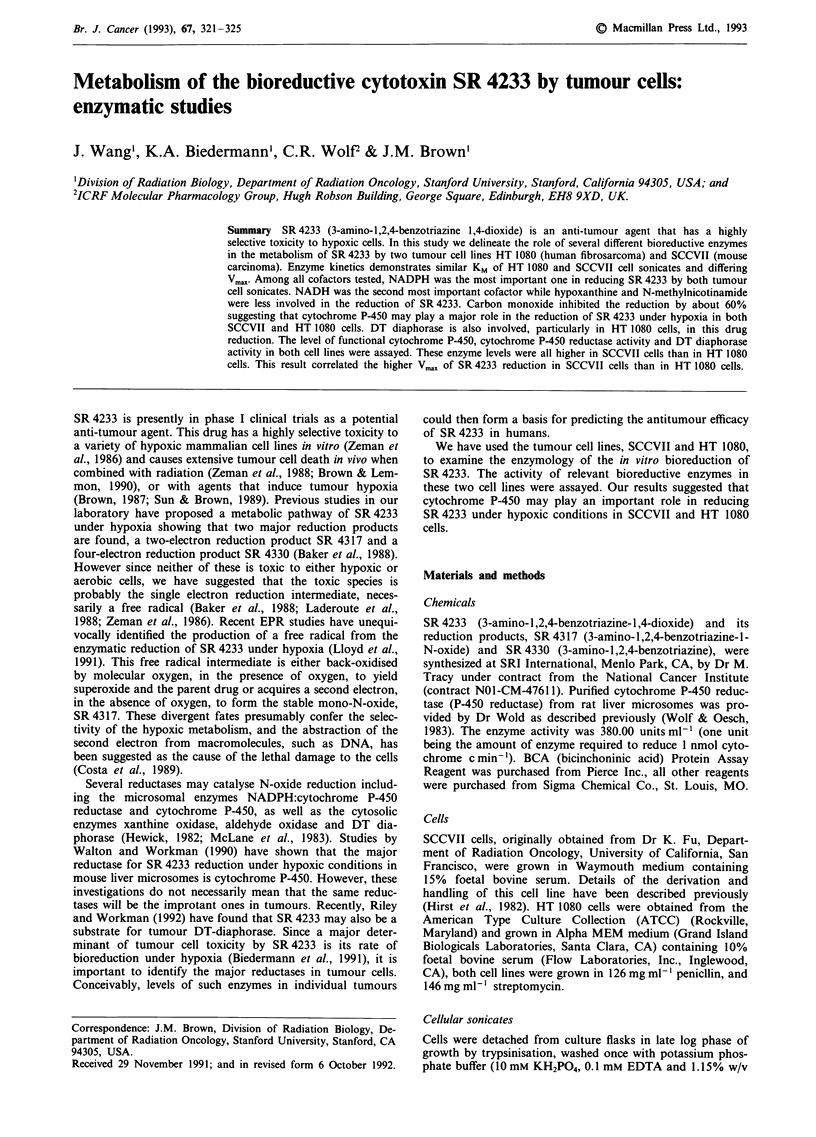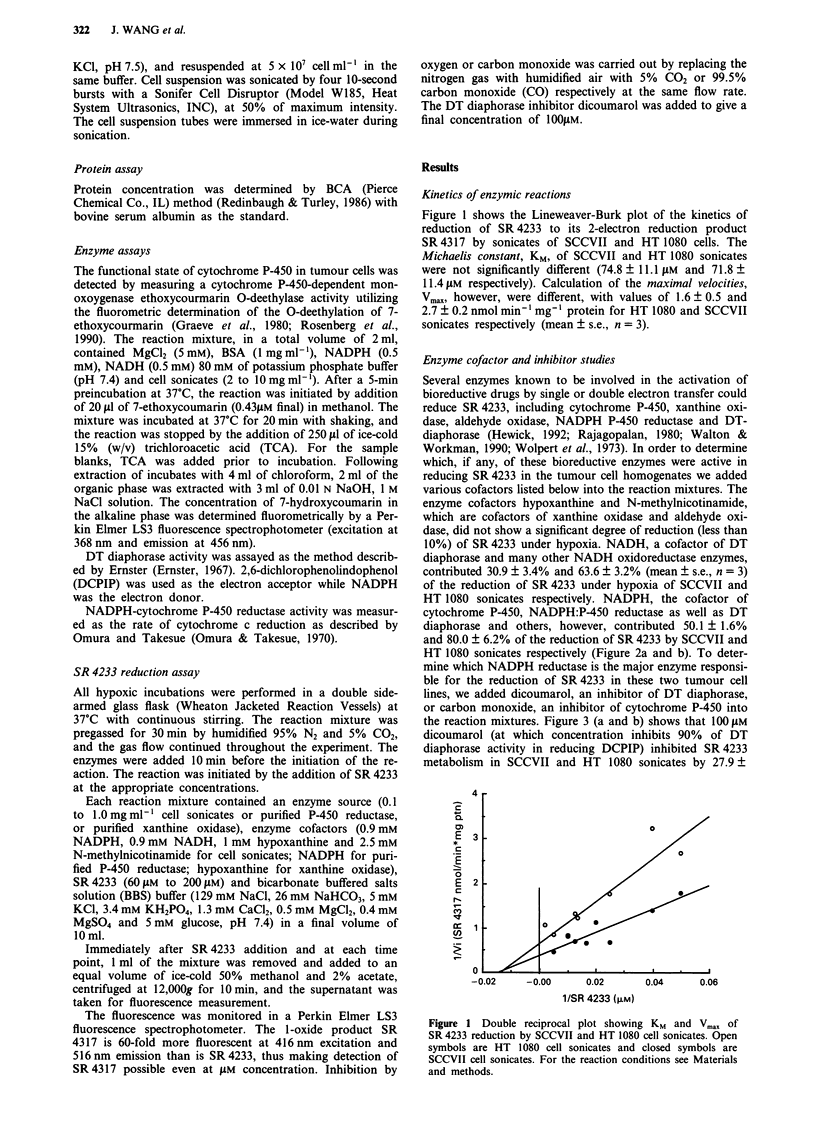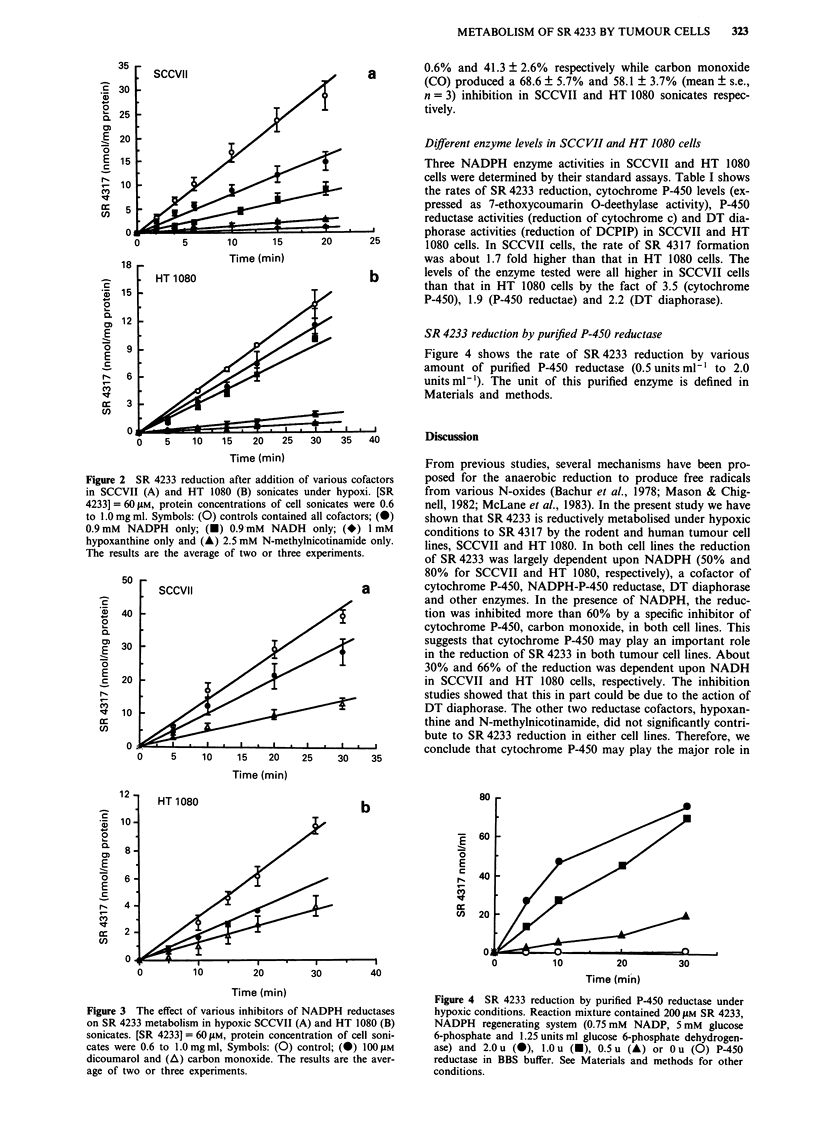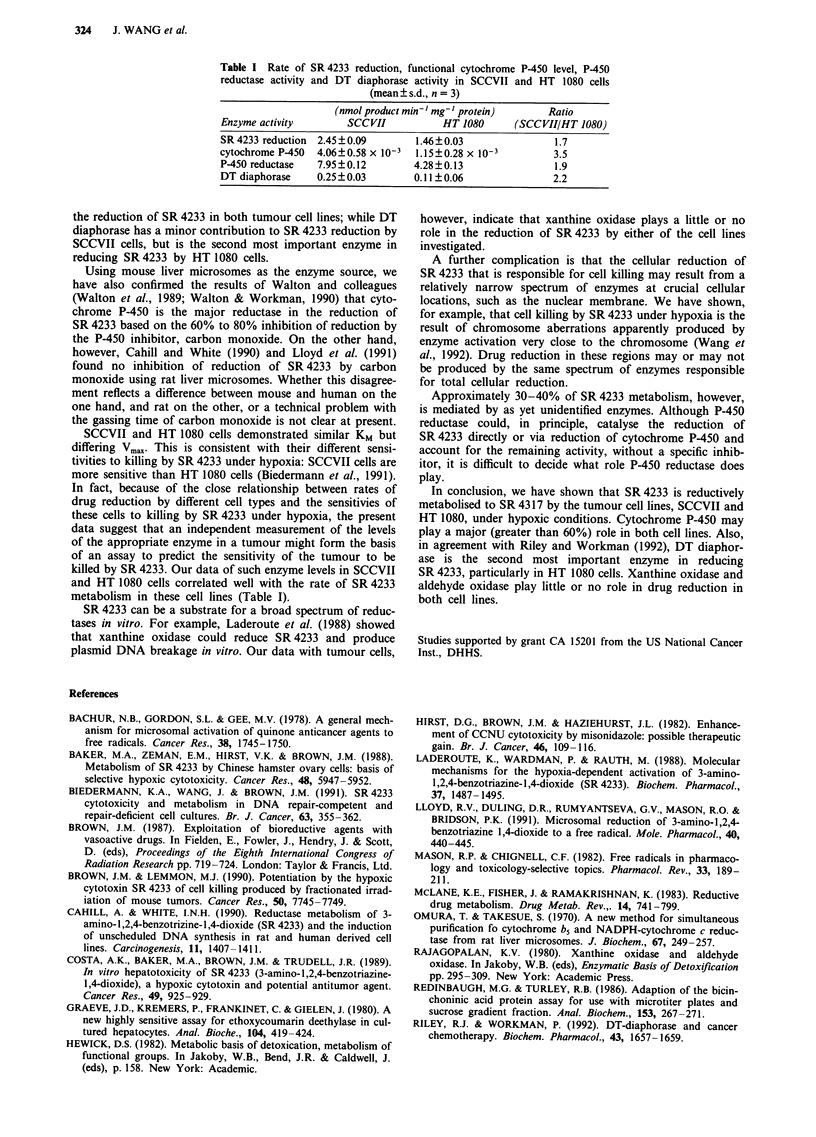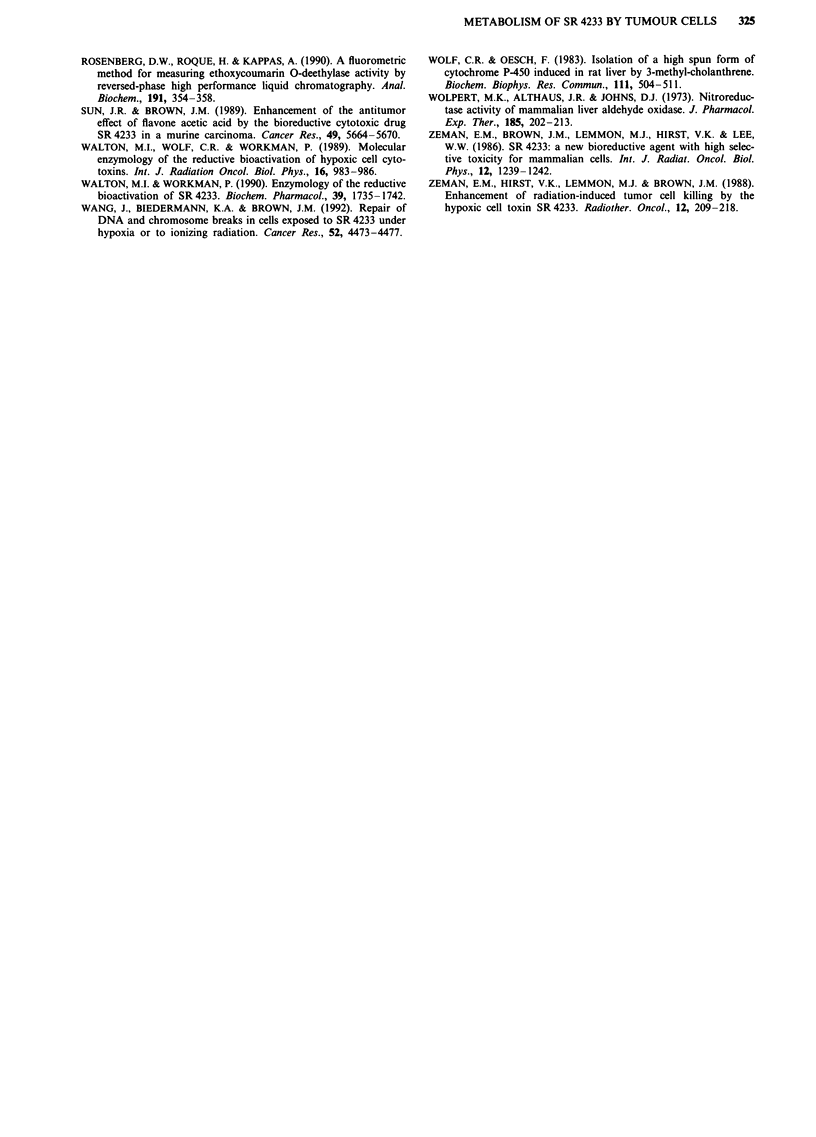# Metabolism of the bioreductive cytotoxin SR 4233 by tumour cells: enzymatic studies.

**DOI:** 10.1038/bjc.1993.59

**Published:** 1993-02

**Authors:** J. Wang, K. A. Biedermann, C. R. Wolf, J. M. Brown

**Affiliations:** Department of Radiation Oncology, Stanford University, California 94305.

## Abstract

SR 4233 (3-amino-1,2,4-benzotriazine 1,4-dioxide) is an anti-tumour agent that has a highly selective toxicity to hypoxic cells. In this study we delineate the role of several different bioreductive enzymes in the metabolism of SR 4233 by two tumour cell lines HT 1080 (human fibrosarcoma) and SCCVII (mouse carcinoma). Enzyme kinetics demonstrates similar KM of HT 1080 and SCCVII cell sonicates and differing Vmax. Among all cofactors tested, NADPH was the most important one in reducing SR 4233 by both tumour cell sonicates. NADH was the second most important cofactor while hypoxanthine and N-methylnicotinamide were less involved in the reduction of SR 4233. Carbon monoxide inhibited the reduction by about 60% suggesting that cytochrome P-450 may play a major role in the reduction of SR 4233 under hypoxia in both SCCVII and HT 1080 cells. DT diaphorase is also involved, particularly in HT 1080 cells, in this drug reduction. The level of functional cytochrome P-450, cytochrome P-450 reductase activity and DT diaphorase activity in both cell lines were assayed. These enzyme levels were all higher in SCCVII cells than in HT 1080 cells. This result correlated the higher Vmax of SR 4233 reduction in SCCVII cells than in HT 1080 cells.


Br. J. Cancer (1993), 67, 321–325 © Macmillan Press Ltd., 1993

### Metabolism of the bioreductive cytotoxin SR 4233 by tumour cells: enzymatic studies

J. Wang[1], K.A. Biedermann[1], C.R. Wolf[2] & J.M. Brown[1]

[1]*Division of Radiation Biology, Department of Radiation Oncology, Stanford University, Stanford, California 94305, USA; and* [2]*ICRF Molecular Pharmacology Group, Hugh Robson Building, George Square, Edinburgh, EH8 9XD, UK.*

**Summary** SR 4233 (3-amino-1,2,4-benzotriazine 1,4-dioxide) is an anti-tumour agent that has a highly selective toxicity to hypoxic cells. In this study we delineate the role of several different bioreductive enzymes in the metabolism of SR 4233 by two tumour cell lines HT 1080 (human fibrosarcoma) and SCCVII (mouse carcinoma). Enzyme kinetics demonstrates similar $K_M$ of HT 1080 and SCCVII cell sonicates and differing $V_{max}$. Among all cofactors tested, NADPH was the most important one in reducing SR 4233 by both tumour cell sonicates. NADH was the second most important cofactor while hypoxanthine and N-methylnicotinamide were less involved in the reduction of SR 4233. Carbon monoxide inhibited the reduction by about 60% suggesting that cytochrome P-450 may play a major role in the reduction of SR 4233 under hypoxia in both SCCVII and HT 1080 cells. DT diaphorase is also involved, particularly in HT 1080 cells, in this drug reduction. The level of functional cytochrome P-450, cytochrome P-450 reductase activity and DT diaphorase activity in both cell lines were assayed. These enzyme levels were all higher in SCCVII cells than in HT 1080 cells. This result correlated the higher $V_{max}$ of SR 4233 reduction in SCCVII cells than in HT 1080 cells.

SR 4233 is presently in phase I clinical trials as a potential anti-tumour agent. This drug has a highly selective toxicity to a variety of hypoxic mammalian cell lines *in vitro* (Zeman *et al.*, 1986) and causes extensive tumour cell death *in vivo* when combined with radiation (Zeman *et al.*, 1988; Brown & Lemmon, 1990), or with agents that induce tumour hypoxia (Brown, 1987; Sun & Brown, 1989). Previous studies in our laboratory have proposed a metabolic pathway of SR 4233 under hypoxia showing that two major reduction products are found, a two-electron reduction product SR 4317 and a four-electron reduction product SR 4330 (Baker *et al.*, 1988). However since neither of these is toxic to either hypoxic or aerobic cells, we have suggested that the toxic species is probably the single electron reduction intermediate, necessarily a free radical (Baker *et al.*, 1988; Laderoute *et al.*, 1988; Zeman *et al.*, 1986). Recent EPR studies have unequivocally identified the production of a free radical from the enzymatic reduction of SR 4233 under hypoxia (Lloyd *et al.*, 1991). This free radical intermediate is either back-oxidised by molecular oxygen, in the presence of oxygen, to yield superoxide and the parent drug or acquires a second electron, in the absence of oxygen, to form the stable mono-N-oxide, SR 4317. These divergent fates presumably confer the selectivity of the hypoxic metabolism, and the abstraction of the second electron from macromolecules, such as DNA, has been suggested as the cause of the lethal damage to the cells (Costa *et al.*, 1989).

Several reductases may catalyse N-oxide reduction including the microsomal enzymes NADPH:cytochrome P-450 reductase and cytochrome P-450, as well as the cytosolic enzymes xanthine oxidase, aldehyde oxidase and DT diaphorase (Hewick, 1982; McLane *et al.*, 1983). Studies by Walton and Workman (1990) have shown that the major reductase for SR 4233 reduction under hypoxic conditions in mouse liver microsomes is cytochrome P-450. However, these investigations do not necessarily mean that the same reductases will be the improtant ones in tumours. Recently, Riley and Workman (1992) have found that SR 4233 may also be a substrate for tumour DT-diaphorase. Since a major determinant of tumour cell toxicity by SR 4233 is its rate of bioreduction under hypoxia (Biedermann *et al.*, 1991), it is important to identify the major reductases in tumour cells. Conceivably, levels of such enzymes in individual tumours could then form a basis for predicting the antitumour efficacy of SR 4233 in humans.

We have used the tumour cell lines, SCCVII and HT 1080, to examine the enzymology of the *in vitro* bioreduction of SR 4233. The activity of relevant bioreductive enzymes in these two cell lines were assayed. Our results suggested that cytochrome P-450 may play an important role in reducing SR 4233 under hypoxic conditions in SCCVII and HT 1080 cells.

Correspondence: J.M. Brown, Division of Radiation Biology, Department of Radiation Oncology, Stanford University, Stanford, CA 94305, USA.
Received 29 November 1991; and in revised form 6 October 1992.

### Materials and methods

### *Chemicals*

SR 4233 (3-amino-1,2,4-benzotriazine-1,4-dioxide) and its reduction products, SR 4317 (3-amino-1,2,4-benzotriazine-1-N-oxide) and SR 4330 (3-amino-1,2,4-benzotriazine), were synthesized at SRI International, Menlo Park, CA, by Dr M. Tracy under contract from the National Cancer Institute (contract N01-CM-47611). Purified cytochrome P-450 reductase (P-450 reductase) from rat liver microsomes was provided by Dr Wold as described previously (Wolf & Oesch, 1983). The enzyme activity was 380.00 units $ml^{-1}$ (one unit being the amount of enzyme required to reduce 1 nmol cytochrome c $min^{-1}$). BCA (bicinchoninic acid) Protein Assay Reagent was purchased from Pierce Inc., all other reagents were purchased from Sigma Chemical Co., St. Louis, MO.

### *Cells*

SCCVII cells, originally obtained from Dr K. Fu, Department of Radiation Oncology, University of California, San Francisco, were grown in Waymouth medium containing 15% foetal bovine serum. Details of the derivation and handling of this cell line have been described previously (Hirst *et al.*, 1982). HT 1080 cells were obtained from the American Type Culture Collection (ATCC) (Rockville, Maryland) and grown in Alpha MEM medium (Grand Island Biologicals Laboratories, Santa Clara, CA) containing 10% foetal bovine serum (Flow Laboratories, Inc., Inglewood, CA), both cell lines were grown in 126 mg $ml^{-1}$ penicllin, and 146 mg $ml^{-1}$ streptomycin.

### *Cellular sonicates*

Cells were detached from culture flasks in late log phase of growth by trypsinisation, washed once with potassium phosphate buffer (10 mM $KH_2PO_4$, 0.1 mM EDTA and 1.15% w/v

KCl, pH 7.5), and resuspended at $5 \times 10^7$ cell $ml^{-1}$ in the same buffer. Cell suspension was sonicated by four 10-second bursts with a Sonifer Cell Disruptor (Model W185, Heat System Ultrasonics, INC), at 50% of maximum intensity. The cell suspension tubes were immersed in ice-water during sonication.

*Protein assay*

Protein concentration was determined by BCA (Pierce Chemical Co., IL) method (Redinbaugh & Turley, 1986) with bovine serum albumin as the standard.

*Enzyme assays*

The functional state of cytochrome P-450 in tumour cells was detected by measuring a cytochrome P-450-dependent monooxygenase ethoxycourmarin O-deethylase activity utilizing the fluorometric determination of the O-deethylation of 7-ethoxycourmarin (Graeve *et al.*, 1980; Rosenberg *et al.*, 1990). The reaction mixture, in a total volume of 2 ml, contained $MgCl_2$ (5 mM), BSA (1 mg $ml^{-1}$), NADPH (0.5 mM), NADH (0.5 mM) 80 mM of potassium phosphate buffer (pH 7.4) and cell sonicates (2 to 10 mg $ml^{-1}$). After a 5-min preincubation at 37°C, the reaction was initiated by addition of 20 µl of 7-ethoxycoumarin (0.43µM final) in methanol. The mixture was incubated at 37°C for 20 min with shaking, and the reaction was stopped by the addition of 250 µl of ice-cold 15% (w/v) trichloroacetic acid (TCA). For the sample blanks, TCA was added prior to incubation. Following extraction of incubates with 4 ml of chloroform, 2 ml of the organic phase was extracted with 3 ml of 0.01 N NaOH, 1 M NaCl solution. The concentration of 7-hydroxycoumarin in the alkaline phase was determined fluorometrically by a Perkin Elmer LS3 fluorescence spectrophotometer (excitation at 368 nm and emission at 456 nm).

DT diaphorase activity was assayed as the method described by Ernster (Ernster, 1967). 2,6-dichlorophenolindophenol (DCPIP) was used as the electron acceptor while NADPH was the electron donor.

NADPH-cytochrome P-450 reductase activity was measured as the rate of cytochrome c reduction as described by Omura and Takesue (Omura & Takesue, 1970).

*SR 4233 reduction assay*

All hypoxic incubations were performed in a double side-armed glass flask (Wheaton Jacketed Reaction Vessels) at 37°C with continuous stirring. The reaction mixture was pregassed for 30 min by humidified 95% $N_2$ and 5% $CO_2$, and the gas flow continued throughout the experiment. The enzymes were added 10 min before the initiation of the reaction. The reaction was initiated by the addition of SR 4233 at the appropriate concentrations.

Each reaction mixture contained an enzyme source (0.1 to 1.0 mg $ml^{-1}$ cell sonicates or purified P-450 reductase, or purified xanthine oxidase), enzyme cofactors (0.9 mM NADPH, 0.9 mM NADH, 1 mM hypoxanthine and 2.5 mM N-methylnicotinamide for cell sonicates; NADPH for purified P-450 reductase; hypoxanthine for xanthine oxidase), SR 4233 (60 µM to 200 µM) and bicarbonate buffered salts solution (BBS) buffer (129 mM NaCl, 26 mM $NaHCO_3$, 5 mM KCl, 3.4 mM $KH_2PO_4$, 1.3 mM $CaCl_2$, 0.5 mM $MgCl_2$, 0.4 mM $MgSO_4$ and 5 mM glucose, pH 7.4) in a final volume of 10 ml.

Immediately after SR 4233 addition and at each time point, 1 ml of the mixture was removed and added to an equal volume of ice-cold 50% methanol and 2% acetate, centrifuged at 12,000*g* for 10 min, and the supernatant was taken for fluorescence measurement.

The fluorescence was monitored in a Perkin Elmer LS3 fluorescence spectrophotometer. The 1-oxide product SR 4317 is 60-fold more fluorescent at 416 nm excitation and 516 nm emission than is SR 4233, thus making detection of SR 4317 possible even at µM concentration. Inhibition by oxygen or carbon monoxide was carried out by replacing the nitrogen gas with humidified air with 5% $CO_2$ or 99.5% carbon monoxide (CO) respectively at the same flow rate. The DT diaphorase inhibitor dicoumarol was added to give a final concentration of 100µM.

### Results

*Kinetics of enzymic reactions*

Figure 1 shows the Lineweaver-Burk plot of the kinetics of reduction of SR 4233 to its 2-electron reduction product SR 4317 by sonicates of SCCVII and HT 1080 cells. The *Michaelis constant*, $K_M$, of SCCVII and HT 1080 sonicates were not significantly different (74.8 ± 11.1 µM and 71.8 ± 11.4 µM respectively). Calculation of the *maximal velocities*, $V_{max}$, however, were different, with values of 1.6 ± 0.5 and 2.7 ± 0.2 nmol $min^{-1}$ $mg^{-1}$ protein for HT 1080 and SCCVII sonicates respectively (mean ± s.e., $n = 3$).

*Enzyme cofactor and inhibitor studies*

Several enzymes known to be involved in the activation of bioreductive drugs by single or double electron transfer could reduce SR 4233, including cytochrome P-450, xanthine oxidase, aldehyde oxidase, NADPH P-450 reductase and DT-diaphorase (Hewick, 1992; Rajagopalan, 1980; Walton & Workman, 1990; Wolpert *et al.*, 1973). In order to determine which, if any, of these bioreductive enzymes were active in reducing SR 4233 in the tumour cell homogenates we added various cofactors listed below into the reaction mixtures. The enzyme cofactors hypoxanthine and N-methylnicotinamide, which are cofactors of xanthine oxidase and aldehyde oxidase, did not show a significant degree of reduction (less than 10%) of SR 4233 under hypoxia. NADH, a cofactor of DT diaphorase and many other NADH oxidoreductase enzymes, contributed 30.9 ± 3.4% and 63.6 ± 3.2% (mean ± s.e., $n = 3$) of the reduction of SR 4233 under hypoxia of SCCVII and HT 1080 sonicates respectively. NADPH, the cofactor of cytochrome P-450, NADPH:P-450 reductase as well as DT diaphorase and others, however, contributed 50.1 ± 1.6% and 80.0 ± 6.2% of the reduction of SR 4233 by SCCVII and HT 1080 sonicates respectively (Figure 2a and b). To determine which NADPH reductase is the major enzyme responsible for the reduction of SR 4233 in these two tumour cell lines, we added dicoumarol, an inhibitor of DT diaphorase, or carbon monoxide, an inhibitor of cytochrome P-450 into the reaction mixtures. Figure 3 (a and b) shows that 100 µM dicoumarol (at which concentration inhibits 90% of DT diaphorase activity in reducing DCPIP) inhibited SR 4233 metabolism in SCCVII and HT 1080 sonicates by 27.9 ±

**Figure 1** Double reciprocal plot showing $K_M$ and $V_{max}$ of SR 4233 reduction by SCCVII and HT 1080 cell sonicates. Open symbols are HT 1080 cell sonicates and closed symbols are SCCVII cell sonicates. For the reaction conditions see Materials and methods.

**Figure 2** SR 4233 reduction after addition of various cofactors in SCCVII (A) and HT 1080 (B) sonicates under hypoxi. [SR 4233] = 60 μM, protein concentrations of cell sonicates were 0.6 to 1.0 mg ml. Symbols: (○) controls contained all cofactors; (●) 0.9 mM NADPH only; (■) 0.9 mM NADH only; (◆) 1 mM hypoxanthine only and (▲) 2.5 mM N-methylnicotinamide only. The results are the average of two or three experiments.

**Figure 3** The effect of various inhibitors of NADPH reductases on SR 4233 metabolism in hypoxic SCCVII (A) and HT 1080 (B) sonicates. [SR 4233] = 60 μM, protein concentration of cell sonicates were 0.6 to 1.0 mg ml, Symbols: (○) control; (●) 100 μM dicoumarol and (△) carbon monoxide. The results are the average of two or three experiments.

0.6% and 41.3 ± 2.6% respectively while carbon monoxide (CO) produced a 68.6 ± 5.7% and 58.1 ± 3.7% (mean ± s.e., $n = 3$) inhibition in SCCVII and HT 1080 sonicates respectively.

### *Different enzyme levels in SCCVII and HT 1080 cells*

Three NADPH enzyme activities in SCCVII and HT 1080 cells were determined by their standard assays. Table I shows the rates of SR 4233 reduction, cytochrome P-450 levels (expressed as 7-ethoxycoumarin O-deethylase activity), P-450 reductase activities (reduction of cytochrome c) and DT diaphorase activities (reduction of DCPIP) in SCCVII and HT 1080 cells. In SCCVII cells, the rate of SR 4317 formation was about 1.7 fold higher than that in HT 1080 cells. The levels of the enzyme tested were all higher in SCCVII cells than that in HT 1080 cells by the fact of 3.5 (cytochrome P-450), 1.9 (P-450 reductae) and 2.2 (DT diaphorase).

### *SR 4233 reduction by purified P-450 reductase*

Figure 4 shows the rate of SR 4233 reduction by various amount of purified P-450 reductase (0.5 units $ml^{-1}$ to 2.0 units $ml^{-1}$). The unit of this purified enzyme is defined in Materials and methods.

### Discussion

From previous studies, several mechanisms have been proposed for the anaerobic reduction to produce free radicals from various N-oxides (Bachur *et al.*, 1978; Mason & Chignell, 1982; McLane *et al.*, 1983). In the present study we have shown that SR 4233 is reductively metabolised under hypoxic conditions to SR 4317 by the rodent and human tumour cell lines, SCCVII and HT 1080. In both cell lines the reduction of SR 4233 was largely dependent upon NADPH (50% and 80% for SCCVII and HT 1080, respectively), a cofactor of cytochrome P-450, NADPH-P-450 reductase, DT diaphorase and other enzymes. In the presence of NADPH, the reduction was inhibited more than 60% by a specific inhibitor of cytochrome P-450, carbon monoxide, in both cell lines. This suggests that cytochrome P-450 may play an important role in the reduction of SR 4233 in both tumour cell lines. About 30% and 66% of the reduction was dependent upon NADH in SCCVII and HT 1080 cells, respectively. The inhibition studies showed that this in part could be due to the action of DT diaphorase. The other two reductase cofactors, hypoxanthine and N-methylnicotinamide, did not significantly contribute to SR 4233 reduction in either cell lines. Therefore, we conclude that cytochrome P-450 may play the major role in

**Figure 4** SR 4233 reduction by purified P-450 reductase under hypoxic conditions. Reaction mixture contained 200 μM SR 4233, NADPH regenerating system (0.75 mM NADP, 5 mM glucose 6-phosphate and 1.25 units ml glucose 6-phosphate dehydrogenase) and 2.0 u (●), 1.0 u (■), 0.5 u (▲) or 0 u (○) P-450 reductase in BBS buffer. See Materials and methods for other conditions.

**Table I** Rate of SR 4233 reduction, functional cytochrome P-450 level, P-450 reductase activity and DT diaphorase activity in SCCVII and HT 1080 cells (mean ± s.d., $n = 3$)

| *Enzyme activity* | *(nmol product min⁻¹ mg⁻¹ protein)* *SCCVII* | *HT 1080* | *Ratio (SCCVII/HT 1080)* |
|---|---|---|---|
| SR 4233 reduction | 2.45 ± 0.09 | 1.46 ± 0.03 | 1.7 |
| cytochrome P-450 | $4.06 \pm 0.58 \times 10^{-3}$ | $1.15 \pm 0.28 \times 10^{-3}$ | 3.5 |
| P-450 reductase | 7.95 ± 0.12 | 4.28 ± 0.13 | 1.9 |
| DT diaphorase | 0.25 ± 0.03 | 0.11 ± 0.06 | 2.2 |

the reduction of SR 4233 in both tumour cell lines; while DT diaphorase has a minor contribution to SR 4233 reduction by SCCVII cells, but is the second most important enzyme in reducing SR 4233 by HT 1080 cells.

Using mouse liver microsomes as the enzyme source, we have also confirmed the results of Walton and colleagues (Walton *et al.*, 1989; Walton & Workman, 1990) that cytochrome P-450 is the major reductase in the reduction of SR 4233 based on the 60% to 80% inhibition of reduction by the P-450 inhibitor, carbon monoxide. On the other hand, however, Cahill and White (1990) and Lloyd *et al.* (1991) found no inhibition of reduction of SR 4233 by carbon monoxide using rat liver microsomes. Whether this disagreement reflects a difference between mouse and human on the one hand, and rat on the other, or a technical problem with the gassing time of carbon monoxide is not clear at present.

SCCVII and HT 1080 cells demonstrated similar $K_M$ but differing $V_{max}$. This is consistent with their different sensitivities to killing by SR 4233 under hypoxia: SCCVII cells are more sensitive than HT 1080 cells (Biedermann *et al.*, 1991). In fact, because of the close relationship between rates of drug reduction by different cell types and the sensitivies of these cells to killing by SR 4233 under hypoxia, the present data suggest that an independent measurement of the levels of the appropriate enzyme in a tumour might form the basis of an assay to predict the sensitivity of the tumour to be killed by SR 4233. Our data of such enzyme levels in SCCVII and HT 1080 cells correlated well with the rate of SR 4233 metabolism in these cell lines (Table I).

SR 4233 can be a substrate for a broad spectrum of reductases *in vitro*. For example, Laderoute *et al.* (1988) showed that xanthine oxidase could reduce SR 4233 and produce plasmid DNA breakage *in vitro*. Our data with tumour cells, however, indicate that xanthine oxidase plays a little or no role in the reduction of SR 4233 by either of the cell lines investigated.

A further complication is that the cellular reduction of SR 4233 that is responsible for cell killing may result from a relatively narrow spectrum of enzymes at crucial cellular locations, such as the nuclear membrane. We have shown, for example, that cell killing by SR 4233 under hypoxia is the result of chromosome aberrations apparently produced by enzyme activation very close to the chromosome (Wang *et al.*, 1992). Drug reduction in these regions may or may not be produced by the same spectrum of enzymes responsible for total cellular reduction.

Approximately 30–40% of SR 4233 metabolism, however, is mediated by as yet unidentified enzymes. Although P-450 reductase could, in principle, catalyse the reduction of SR 4233 directly or via reduction of cytochrome P-450 and account for the remaining activity, without a specific inhibitor, it is difficult to decide what role P-450 reductase does play.

In conclusion, we have shown that SR 4233 is reductively metabolised to SR 4317 by the tumour cell lines, SCCVII and HT 1080, under hypoxic conditions. Cytochrome P-450 may play a major (greater than 60%) role in both cell lines. Also, in agreement with Riley and Workman (1992), DT diaphorase is the second most important enzyme in reducing SR 4233, particularly in HT 1080 cells. Xanthine oxidase and aldehyde oxidase play little or no role in drug reduction in both cell lines.

Studies supported by grant CA 15201 from the US National Cancer Inst., DHHS.

### References

BACHUR, N.B., GORDON, S.L. & GEE, M.V. (1978). A general mechanism for microsomal activation of quinone anticancer agents to free radicals. *Cancer Res.*, **38**, 1745–1750.

BAKER, M.A., ZEMAN, E.M., HIRST, V.K. & BROWN, J.M. (1988). Metabolism of SR 4233 by Chinese hamster ovary cells: basis of selective hypoxic cytotoxicity. *Cancer Res.*, **48**, 5947–5952.

BIEDERMANN, K.A., WANG, J. & BROWN, J.M. (1991). SR 4233 cytotoxicity and metabolism in DNA repair-competent and repair-deficient cell cultures. *Br. J. Cancer*, **63**, 355–362.

BROWN, J.M. (1987). Exploitation of bioreductive agents with vasoactive drugs. In Fielden, E., Fowler, J., Hendry, J. & Scott, D. (eds), *Proceedings of the Eighth International Congress of Radiation Research* pp. 719–724. London: Taylor & Francis, Ltd.

BROWN, J.M. & LEMMON, M.J. (1990). Potentiation by the hypoxic cytotoxin SR 4233 of cell killing produced by fractionated irradiation of mouse tumors. *Cancer Res.*, **50**, 7745–7749.

CAHILL, A. & WHITE, I.N.H. (1990). Reductase metabolism of 3-amino-1,2,4-benzotrizine-1,4-dioxide (SR 4233) and the induction of unscheduled DNA synthesis in rat and human derived cell lines. *Carcinogenesis*, **11**, 1407–1411.

COSTA, A.K., BAKER, M.A., BROWN, J.M. & TRUDELL, J.R. (1989). *In vitro* hepatotoxicity of SR 4233 (3-amino-1,2,4-benzotriazine-1,4-dioxide), a hypoxic cytotoxin and potential antitumor agent. *Cancer Res.*, **49**, 925–929.

GRAEVE, J.D., KREMERS, P., FRANKINET, C. & GIELEN, J. (1980). A new highly sensitive assay for ethoxycoumarin deethylase in cultured hepatocytes. *Anal. Bioche.*, **104**, 419–424.

HEWICK, D.S. (1982). Metabolic basis of detoxication, metabolism of functional groups. In Jakoby, W.B., Bend, J.R. & Caldwell, J. (eds), p. 158. New York: Academic.

HIRST, D.G., BROWN, J.M. & HAZIEHURST, J.L. (1982). Enhancement of CCNU cytotoxicity by misonidazole: possible therapeutic gain. *Br. J. Cancer*, **46**, 109–116.

LADEROUTE, K., WARDMAN, P. & RAUTH, M. (1988). Molecular mechanisms for the hypoxia-dependent activation of 3-amino-1,2,4-benzotriazine-1,4-dioxide (SR 4233). *Biochem. Pharmacol.*, **37**, 1487–1495.

LLOYD, R.V., DULING, D.R., RUMYANTSEVA, G.V., MASON, R.O. & BRIDSON, P.K. (1991). Microsomal reduction of 3-amino-1,2,4-benzotriazine 1,4-dioxide to a free radical. *Mole. Pharmacol.*, **40**, 440–445.

MASON, R.P. & CHIGNELL, C.F. (1982). Free radicals in pharmacology and toxicology-selective topics. *Pharmacol. Rev.*, **33**, 189–211.

MCLANE, K.E., FISHER, J. & RAMAKRISHNAN, K. (1983). Reductive drug metabolism. *Drug Metab. Rev.*,. **14**, 741–799.

OMURA, T. & TAKESUE, S. (1970). A new method for simultaneous purification fo cytochrome $b_5$ and NADPH-cytochrome *c* reductase from rat liver microsomes. *J. Biochem.*, **67**, 249–257.

RAJAGOPALAN, K.V. (1980). Xanthine oxidase and aldehyde oxidase. In Jakoby, W.B. (eds), *Enzymatic Basis of Detoxification* pp. 295–309. New York: Academic Press.

REDINBAUGH, M.G. & TURLEY, R.B. (1986). Adaption of the bicinchoninic acid protein assay for use with microtiter plates and sucrose gradient fraction. *Anal. Biochem.*, **153**, 267–271.

RILEY, R.J. & WORKMAN, P. (1992). DT-diaphorase and cancer chemotherapy. *Biochem. Pharmacol.*, **43**, 1657–1659.

ROSENBERG, D.W., ROQUE, H. & KAPPAS, A. (1990). A fluorometric method for measuring ethoxycoumarin O-deethylase activity by reversed-phase high performance liquid chromatography. *Anal. Biochem.*, **191**, 354–358.

SUN, J.R. & BROWN, J.M. (1989). Enhancement of the antitumor effect of flavone acetic acid by the bioreductive cytotoxic drug SR 4233 in a murine carcinoma. *Cancer Res.*, **49**, 5664–5670.

WALTON, M.I., WOLF, C.R. & WORKMAN, P. (1989). Molecular enzymology of the reductive bioactivation of hypoxic cell cytotoxins. *Int. J. Radiation Oncol. Biol. Phys.*, **16**, 983–986.

WALTON, M.I. & WORKMAN, P. (1990). Enzymology of the reductive bioactivation of SR 4233. *Biochem. Pharmacol.*, **39**, 1735–1742.

WANG, J., BIEDERMANN, K.A. & BROWN, J.M. (1992). Repair of DNA and chromosome breaks in cells exposed to SR 4233 under hypoxia or to ionizing radiation. *Cancer Res.*, **52**, 4473–4477.

WOLF, C.R. & OESCH, F. (1983). Isolation of a high spun form of cytochrome P-450 induced in rat liver by 3-methyl-cholanthrene. *Biochem. Biophys. Res. Commun.*, **111**, 504–511.

WOLPERT, M.K., ALTHAUS, J.R. & JOHNS, D.J. (1973). Nitroreductase activity of mammalian liver aldehyde oxidase. *J. Pharmacol. Exp. Ther.*, **185**, 202–213.

ZEMAN, E.M., BROWN, J.M., LEMMON, M.J., HIRST, V.K. & LEE, W.W. (1986). SR 4233: a new bioreductive agent with high selective toxicity for mammalian cells. *Int. J. Radiat. Oncol. Biol. Phys.*, **12**, 1239–1242.

ZEMAN, E.M., HIRST, V.K., LEMMON, M.J. & BROWN, J.M. (1988). Enhancement of radiation-induced tumor cell killing by the hypoxic cell toxin SR 4233. *Radiother. Oncol.*, **12**, 209–218.